# The “SleepWell” intervention for patients with insomnia and persistent pain: a study protocol for a randomised waiting-list controlled trial of a cognitive behavioural group therapy programme

**DOI:** 10.1186/s13063-025-09041-z

**Published:** 2025-08-27

**Authors:** Jan H. Rosenvinge, Svein Bergvik, Karin Abeler, Kristin Tvedt, Torkil Berge, Nina Lang, Maja Wilhelmsen, Lena Danielsson, Gunn Pettersen, Oddgeir Friborg

**Affiliations:** 1 Faculty of Health Sciences, Department of Psychology, UiT - The Arctic University of Norway, 9037 Tromsø, Norway; 2 Department of Neurology and Neurophysiology, University Hospital of North Norway, 9038 Tromsø, Norway; 3 Diakonhjemmet Hospital, Oslo, Norway; 4 Department of Rehabilitation, University Hospital of North Norway, 9038 Tromsø, Norway; 5 Faculty of Health Sciences, Department of Clinical Medicine, UiT - The Arctic University of Norway, 9037 Tromsø, Norway; 6 Department of Pain Management, University Hospital of North Norway, 9038 Tromsø, Norway; 7 Faculty of Health Sciences, Department of Health and Caring Sciences, UiT – The Arctic University of Norway, Tromsø, Norway

**Keywords:** Pain, Comorbid insomnia, SleepWell intervention, Randomised controlled trial, Waiting list design, Polysomnography, Actigraphy, Sleep questionnaires

## Abstract

**Background:**

Patients with persistent pain and comorbid insomnia often experience a dual burden with significant day- and nighttime impairments. This comorbidity is associated with health problems like depression and a self-perpetuating vicious circle in which pain and insomnia symptoms mutually reinforce each other. Cognitive behavioural therapy for insomnia (CBT-i) has demonstrated efficacy in facilitating the behavioural and psychological changes necessary to improve sleep. However, its applicability to patients with the insomnia-pain comorbidity condition is underexplored. We will expand the knowledge base of CBT-i to this patient group by examining the effects on core insomnia symptoms, including sleep onset, the frequency and duration of nightly and early morning awakenings, sleep efficiency and daytime impairments at post-test and follow-up. Secondary outcomes include reductions in pain intensity and interference, depression and fatigue; improved pain acceptance and quality of life; and more adaptive sleep-related beliefs. This study also explores mediators of the expected effects, patient experiences of the feasibility and acceptability of the intervention and how these experiences relate to individual differences in treatment benefits.

**Methods:**

The study includes hospital patients with a chronic pain condition according to the criteria from the International Association for the Study of Pain and a DSM-5 diagnosis of insomnia. The study will recruit 106 patients based on a power analysis that accounts for 20% dropout, and block-randomise them to a group-based CBT-i intervention or treatment as usual (TAU). The latter consists of consultations and potential pain- and sleep medication. The participant timeline includes a baseline registration, seven sessions of the CBT-i within a 10-week period, a post-test and two follow-up measurements at 4 and 12 months, respectively. The statistical analyses will be intention-to-treat and include random factors to adjust for data dependencies. Patients’ experiences of feasibility and acceptability will be analysed using a reflexive thematic approach.

**Discussion:**

This study addresses a knowledge gap by evaluating the effectiveness of CBT-i adapted for patients with insomnia and non-malignant, persistent pain. Given positive findings, the study may support clinical recommendations by providing empirical evidence for implementing psychological sleep interventions for somatic hospital patients having comorbid sleep issues.

**Trial registration:**

Clinical Trials.gov ID NCT06351839. Registered 08 April 2024.

## Background and rationale {6a}

The present study protocol outlines the rationale for a randomised controlled trial (RCT) examining the effectiveness of “Sleep-Well”, a cognitive behavioural therapy for insomnia (CBT-i), to treat comorbid insomnia in patients with persistent pain. The DSM-5 insomnia diagnosis comprises core difficulties with falling asleep, nightly awakenings and early morning awakening without being able to return to sleep [1]. These sleep disturbances must be accompanied by significant distress and daytime impairments in social, occupational, educational or behavioural areas of functioning. Other notable clinical features following non-restorative sleep are cognitive dysfunction (e.g. attention or concentration), fatigue or daytime sleepiness and depressive mood disturbances[2, 3]. The duration (at least 3 months) and frequency requirement (at least 3 days/week) of the core DSM-5 symptoms of insomnia [1] increases the risk of significant health consequences, such as cardiovascular diseases [4] and poorer overall health [5]. The 60–80% comorbidity between persistent, non-malignant pain and sleep problems [6, 7] raises the risk of sleep-stage shifts and reduced slow-wave sleep, resulting in poorer sleep quality [8] as well as a greater pain severity, longer pain durations and higher levels of disability [9]. Pain perceptions may disrupt sleep, and insomnia can exacerbate these perceptions. A stronger path from nightly poor sleep to next-day pain [10–12] than the reverse [13] suggests that pain alleviation could be an additional benefit from sleep interventions.

A review by Haack et al. [14] discusses numerous neurobiological etiological factors, e.g. opioids, adenosine signalling, cortisol, immune responses, melatonin and monoaminergic transmitters (e.g. serotonin, norepinephrine and dopamine) in the regulation of sleep and pain, which for several of these, also includes depression [13]. It has been argued that insomnia is a stronger risk factor for depression than vice versa [15]. However, evidence from large population-based datasets [16] strengthens the idea of a reciprocal relationship. In a prospective study, O’Brien et al. [17] identified depression as mediating the relationship between sleep disturbances and pain. Regardless, the interconnections between sleep, pain and depression are substantial [7], implying that effective treatment of insomnia also may alleviate pain [18] and depression [19].

Shared cognitive and emotional factors for sleep and pain comprise repetitive negative thinking. For example, patients may experience persistent, uncontrollable rumination about past events and anticipatory worry, as well as catastrophic thinking about the consequences of persistent pain and sleep loss [20]. Dysfunctional sleep beliefs and selective attention to incidents supporting these beliefs generally exacerbate disturbed sleep. Sleep-related worry associated with safety behaviours aimed at avoiding the feared consequences of sleeplessness may initiate a vicious cycle that, along with physiological and cognitive arousal, maintains insomnia [21]. A systematic review and meta-analysis by Bilterys et al. [22] found a strong odds ratio (7.6) for developing insomnia among pain patients who display catastrophic thinking. This vicious cycle may, in turn, exacerbate autonomic activation at bedtime, potentially contributing to elevated cortisol levels in pain patients [23], reduced restorative slow-wave sleep [24] and diminished response to pain treatment [25].

The marginal and sometimes iatrogenic effects of sleep medications in treating sleep disturbances and partly pain symptoms [26–28] underscore the need for psychological approaches, and CBT-i is the recommended first-line treatment in the USA [29], Europe [30] and Norway [31], where the current study is situated. There is strong evidence for the effects of CBT-i on self-reported enduring insomnia, but weaker evidence for objective markers of sleep onset and sleep efficiency [32]. Reviews and meta-analyses [33, 34] indicate that CBT-i improves nighttime symptoms like sleep onset, sleep continuity and sleep efficiency, which benefit daytime functioning [35] and may reduce reliance on sleep medication [36]. From a cost–benefit perspective, group therapy formats may be preferable as group-based CBT-i seems equally effective as individual CBT-i [35].

For insomnia comorbid with a pain condition, several reviews and meta-analyses of RCT studies [18, 37, 38] indicate that CBT-i is highly effective in reducing insomnia symptoms, but shows weaker and inconsistent effects on pain-related symptoms. Similarly, treatment studies with patients having a broader spectrum of somatic and mental health issues report substantial improvements in self-reported insomnia, while objective sleep indices (e.g. actigraphy) show more modest gains [39]. The impact of CBT-i on objective sleep measures, particularly polysomnographic (PSG) variables, is generally less robust than its effects on subjective sleep reports, although the alignment between subjective and objective total sleep time (TST) may be somewhat better [40]. A known feature of insomnia is the misperception of sleep, where patients often underestimate sleep duration and overestimate sleep onset latency or the frequency and duration of awakenings [41–44]. Consequently, treatment studies frequently find large improvements in subjective sleep evaluations that are not reflected in objective data from actigraphy or PSG [40]. This discrepancy likely reflects a core clinical feature of insomnia rather than a methodological flaw, as diagnosis is based on subjective reports without requiring objective confirmation. Understanding the mechanisms behind this mismatch may have diagnostic and clinical relevance, particularly since greater discrepancies are associated with poorer overall functioning [44]. Contributing factors to this misperception include heightened mental distress, adverse life events, worry, selective attention, psychophysiological arousal [44] and depressed mood [45]. The present study incorporates a broad set of behavioural, cognitive and affective measures, enabling exploration of the most salient contributors to sleep misperception.

The behavioural components of CBT-i manuals converge around sleep hygiene education, sleep restriction, relaxation training and stimulus control procedures to strengthen the patients’ association between bed/bedroom and sleep [46]. Stimulus control, particularly when combined with sleep restriction and consistent bedtime routines, has been shown to be essential for achieving positive sleep outcomes [46–49]. Numerous conceptualisations of the cognitive components in insomnia treatment have been proposed [47], as changing negative cognitions about sleep is considered a central mechanism underlying CBT-i’s effectiveness [50, 51]. The present study relies predominantly on a cognitive model developed by Harvey and colleagues [42] focusing on reducing the insomnia-maintaining factors, i.e. ruminative worry (contributing to cognitive arousal), selective attention and monitoring, misperceptions of sleep and daytime consequences, dysfunctional beliefs about sleep and counterproductive avoidance behaviours.

The empirical support for the cognitive elements of CBT-i has, however, been less consistent. A recent meta-analysis [52] showed that CBT-i effectively reduced worry, but not rumination.

Since rumination is a powerful depressogenic behaviour [53] that may prolong the duration of negative mood states and interfere with problem-solving and instrumental behaviours, it represents a trans-diagnostic mental health vulnerability factor that may exacerbate and maintain physiological stress responses despite psychological support [54]. Rather than challenging the content validity of cognitions, a meta-cognitive CBT-i approach questions the assumptions of the benefits of worrying, selective attention to pre-sleep insomnia indications and ruminations about daytime or health consequences. Since this may be a central cognitive feature that maintains insomnia [55], as well as a trans-diagnostic factor, the present study may be tenable in treating or preventing relapse in related comorbidities, such as depression and anxiety [56, 57].

The rationale for conducting the present study is to investigate the effects of a CBT-i intervention that includes well-documented behavioural, cognitive and metacognitive treatment elements for patients suffering insomnia-pain comorbidity. This is accomplished through a rigorous RCT design and a battery of self-report and objective outcome measures.

### Explanation for the choice of comparators {6b}

Treatment-as-usual (TAU) is used as a comparator to the SleepWell treatment arm. The TAU condition reflects the standard care practice at the study site. TAU is unsystematic in its content and scope, and may involve some rudimentary sleep assessment, essential advice to improve sleep and sleep hygiene and potential prescription of sleep medication on indication, provided by the hospital’s pain clinic or their general practitioner (GP). It represents a semi-active control condition that may adjust expectancy or placebo effects. During the waiting period before entering the “SleepWell” intervention, TAU participants will be surveyed about the health services they received during this time.

#### Study objectives {7}

The primary aim is to examine the effectiveness of a CBT-i treatment manual, the “SleepWell” intervention, compared to TAU. We hypothesise that the “SleepWell” intervention will outperform TAU in reducing insomnia symptoms specified as favourable changes in nocturnal problems (shorter sleep onset, better sleep maintenance, delayed early morning awakening and better sleep efficiency) and daytime function (less dissatisfaction with sleep, and better function in school/work/social life). We also expect larger treatment benefits to be displayed in self-reported than in objectively assessed comparable sleep data.

Secondary aims with a similar hypothesis direction are to examine whether the intervention may cause less depression and anxiety, pain symptoms/regions, fatigue, less use of sleep medication and improvement in quality of life.

Exploratory aims relate to analyses of possible mediators of treatment effects. This will be supplemented by analyses of the feasibility and acceptability of the “SleepWell” intervention using data from qualitative interviews of study completers with varying outcomes as well as of those who drop out or withdraw from the study. Finally, moderator analyses will be conducted to explore possible subgroups that may or may not benefit from the treatment.

#### Trial design {8}

The RCT waiting list design is used to compare the SleepWell intervention to TAU. After being on the WL condition for four months, the TAU patients will cross over to the SleepWell study arm, thus preventing dropouts. To ensure balanced group sizes between the treatment SleepWell intervention and TAU, participants will be block-randomised using variable block sizes of 4, 6 and 8, respectively.

## Methods: patients, interventions and outcomes

This study protocol adheres to the SPIRIT reporting guidelines [58].

### Study setting {9}

Eligible patients will be recruited from the Department of Pain Management and the Department of Physical Medicine and Rehabilitation, both of which are outpatient clinics at the University Hospital of North Norway. Patients may also be recruited from GPs in the city of Tromsø. The intervention will be conducted at the Department of Psychology at the UiT–The Arctic University of Norway.

### Eligibility criteria {10}

Eligible are patients aged 18–75 years with a chronic non-malignant pain condition as defined by the International Association for the Study of Pain (IASP) [59], and who meet the DSM-5 criteria for comorbid insomnia [1] determined by the Duke structured clinical interview [60, 61].

Exclusion criteria are (1) a comorbid drug abuse diagnosis, (2) involvement in ongoing compensation and/or insurance cases related to their current or previous health or treatment, (3) a current medical treatment using over 100 mg oral morphine equivalents, (4) a current involvement in other psychological treatments for pain and/or insomnia, (5) admittance to hospital psychiatric treatment during the past 12 months, (6) an epilepsy diagnosis, (7) poor Norwegian communication skills or (8) suffering from restless legs or sleep apnoea.

### Intervention descriptions {11a}

The group therapy format for the SleepWell intervention will consist of 6–8 patients in each group, led by two therapists, over seven sessions (~ 1.5 h per session). The first six sessions will be conducted weekly. Eight weeks thereafter, the seventh one is delivered as a consolidation session. The intervention follows a Norwegian group-based CBT-i manual for insomnia [62, 63], which is adapted for use on somatic patients. Between-session homework assignments are described in a corresponding paper workbook that is handed out during the first session. The treatment manual incorporates evidence-based therapeutic components commonly used in sleep intervention protocols [46, 47, 51]. The core components include behavioural procedures, i.e. stimulus control and sleep restriction—and cognitive and metacognitive strategies. Patients will also use implementation intentions [64] to change sleep-related habits.

- Part 1 introduces the treatment rationale and methods to achieve the intervention’s overall goals. Patients will be introduced to the self-report tools necessary for the daily self-monitoring assignments, which are expected to be completed between sessions. This module also includes psychoeducation on sleep physiology, causes of sleep problems, circadian rhythms and the rationale and practical usage of sleep diaries for recording sleep timing, continuity and duration.
- Part 2 focuses on establishing good sleep hygiene habits, introducing stimulus control and the practice of sleep restriction and the use of implementation intentions is introduced as a method for changing sleep-related habits.
- Part 3 addresses cognitive and meta-cognitive factors that maintain sleep disorders, and questions patients’ rationale for their attitudes and beliefs about the need for being occupied with sleep-related worry and rumination.
- Part 4 emphasises relapse prevention by integrating more functional sleeping habits into daily routines.

Table 1 summarises the main aims and contents/exercises across the four modules and seven sessions of the SleepWell intervention.

**Table 1:** Overview of the SleepWell intervention [65, 66]

	**Main objective**	**Content and exercises**
**Part 1**	**Understanding sleep problems and assessment of sleep patterns**	
Session 1	Learn about the treatment rationale and the working methods. Provide education about insomnia, instigate motivation, and adherence to the programme. Instill trust in the treatment process	The cognitive approach to insomnia The sleep diary—its purpose Introduction to the SHI-13*
	*Home assignment*: Read the course material about causes of insomnia, and how to use the sleep diary form. Reflect on the nature of day- and nighttime insomnia-maintaining factors, and the possible required behavioral changes	
**Part 2**	**Behavioural strategies**	
Session 2	Stimulus control: Re-associate the bed with sleepiness instead of wakefulness. Understanding sleep habits and how to change them through implementation intentions (action rules). Reinforce beliefs in long-term sleep benefits above immediate comfort	Review of home assignments after session 1. Go to sleep only when sleepy, leave bed if unable to sleep, create action rules (e.g. *If I cannot sleep, then I will leave the bedroom and return only when sleepy*”), and establish consistency in bed- and wakeup schedules
	*Home assignment:* Read the course material about conditional rules, stimulus control, and sleep-affecting substances. Settle on a fixed bedtime schedule. Continue sleep registration in the sleep diary	
Session 3	Introduction to the benefits of restricting time in bed to facilitate sleep accumulation, regular circadian rhythm, and overall sleep quality	Review of home assignments after session 2. Calculate sleep efficiency (SE %) from the sleep diary. Implement sleep restriction by constraining the time in bed based on patients’ sleep timing and efficiency
	*Home assignment*: Continue completing the sleep diary. Maintain stimulus control habits and action rules. Read course material about sleep restriction. Calculate SE (%) after a week’s registration	
**Part 3**	**Cognitive and metacognitive strategies**	
Session 4	Learn about relaxation techniques and mindfulness for assistance in selective attention. Introduction to the supportive inner dialogue perspective	Review of home assignments after session 3. Focus on mindfulness and dialogues about sleep-disrupting cognitions and metacognitions, notably attitudes toward convictions of the need to control worry and catastrophic thoughts about sleep and insomnia (e.g. “*If I worry, then I will remind myself that worry is not something I need to be worried about*”)
	*Home assignment:* Read course material on mental strategies. Complete a questionnaire about attitudes towards sleep and sleep-related worry and rumination. Continue using the sleep diary	
Session 5	Cognitive and metacognitive approaches	Review of home assignments after session 4. Use of distortions and diversions to combat worry and rumination about sleeplessness, mindful attention, and structures in one’s thinking. Practise action rules (e.g. “*If worrying thoughts appear, then I can address them later*”)
	*Home assignment:* Read the course material on how to combat worry, catastrophic thinking, and rumination. Complete the DIRS** and the sleep diary	
**Part 4**	**Summary and the Road Ahead**	
Session 6	Strategies for maintenance and relapse prevention	Review home assignments after session 5. Review methods and strategies used so far. Strengthen the integration of functional sleeping habits in daily life routines
	*Home assignment:* Reflect on the challenges of changing sleeping habits, and what is useful for relapse prevention. Continue to complete the sleep diary, maintain stimulus control and calculate SE %. Reflect on themes suitable for a discussion at the final session	
Session 7	Closing and the way ahead (about 4–6 weeks after session 6)	Status. Discussions about how to maintain and secure further progress

^*^ *SHI*-*13* Sleep Hygiene Index [72]. ** *DIRS* Daytime Insomnia Symptom Response Scale [73]

### Criteria for discontinuing or modifying allocated interventions {11b}

Intervention discontinuation may occur if a patient withdraws consent or if an unforeseen adverse event poses a significant risk to the participant’s well-being or health.

### Strategies to improve adherence to interventions {11c}

All group therapy sessions will be audiotaped to assess the therapists’ compliance with and fidelity to the treatment manual. At the end of each session, the group therapists review the upcoming homework assignments and ensure that the next scheduled session fits all participants. Based on participant consent, therapists are authorised to contact participants if they are absent from a session.

### Relevant concomitant care or prohibited during the trial {11d}

The use of sleep medications is permitted during the entire study period, but participants are requested to refrain from engaging in any new psychological treatments.

### Outcomes {12}

The overall hypothesis is that the SleepWell intervention will outperform TAU. This will be tested using the following primary (A) and secondary (B) outcome measures:

(A)Favourable changes in the DSM-5 insomnia criteria (1) related to nocturnal symptoms (i.e. SOL-sleep onset latency, WASO-wake after sleep onset, EMA-early morning awakening) and daytime functioning (e.g. reduced dissatisfaction with nighttime sleep and fewer functional problems in education, work or social life). This is assessed with different classes of insomnia measures, i.e. the semi-structured Duke clinical interview for insomnia [60, 61], and the DSM-5 adapted Bergen Insomnia Scale (BIS) [67]. Comparable nocturnal improvements in SOL, WASO and EMA are registered across 7 days using an actigraphy recording device, while the sleep diary complements the actigraphy data. The clinical assessment process for diagnosing insomnia is primarily based on patients’ subjective reports. Thus, the SleepWell intervention will be considered efficacious if it results in a significant reduction in self-reported insomnia symptoms, as measured by the BIS, or in the proportion of individuals who satisfy the criteria for an insomnia diagnosis. Concomitant reductions in nocturnal sleep symptoms, as measured with the actigraphy/sleep diary, will broaden the support of the SleepWell.

(B)Improvements in objectively measured sleep with the “Home Sleep Test” device in terms of an increased proportion and duration of slow-wave sleep, reduced sleep fragmentation and fewer sleep disruptions due to EEG-based microarousals. Secondary measures also include reductions in patient-reported depression and anxiety, pain intensity and interference, fatigue and use of sleep medication, and improvements in pain acceptance and quality of life.

### Participant timeline {13}

**Fig. 1:**
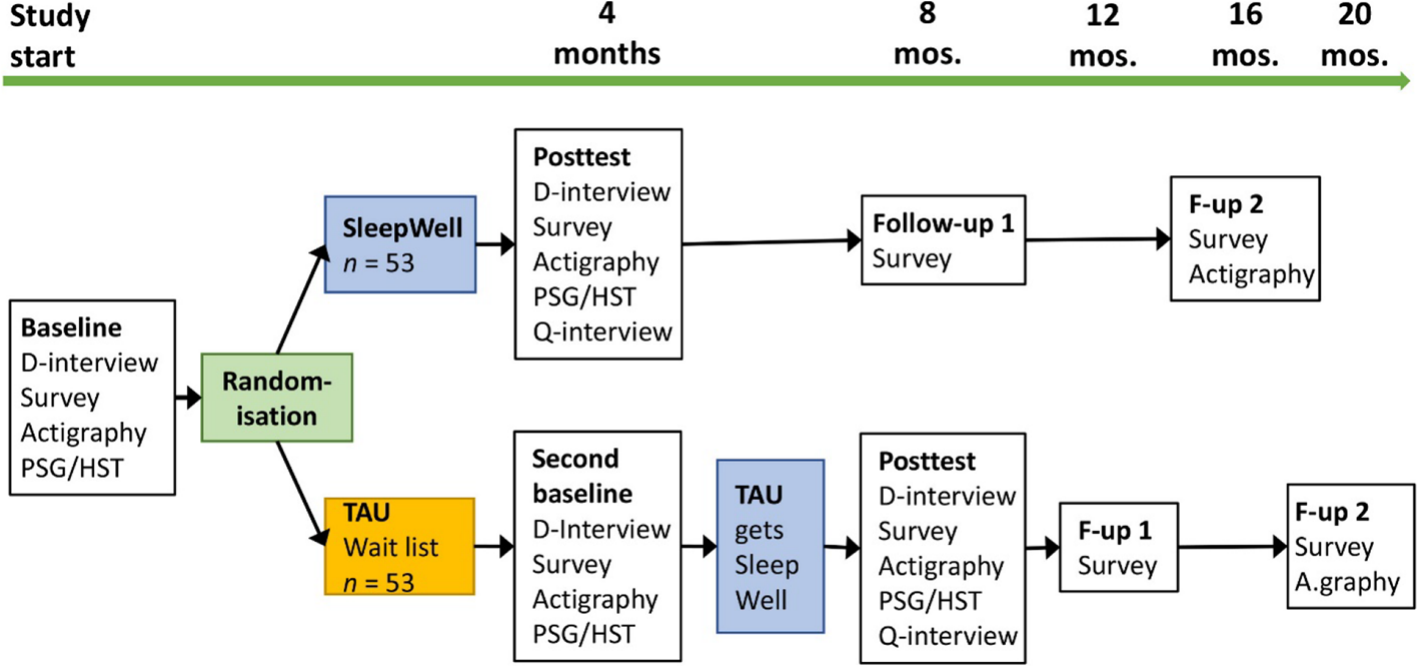
Depiction of study design and timing of assessments. *D*-*interview* Duke diagnostic insomnia interview, *Q*-*interview* Qualitative interviews. Survey = Questionnaire outcome and mediation data according to section *Plans for assessment and collection of outcomes* {18a}. *PSG*/*HST* Polysomnographic Home Sleep Test with EEG-based registering of brain activity during sleep

**Fig. 2:**
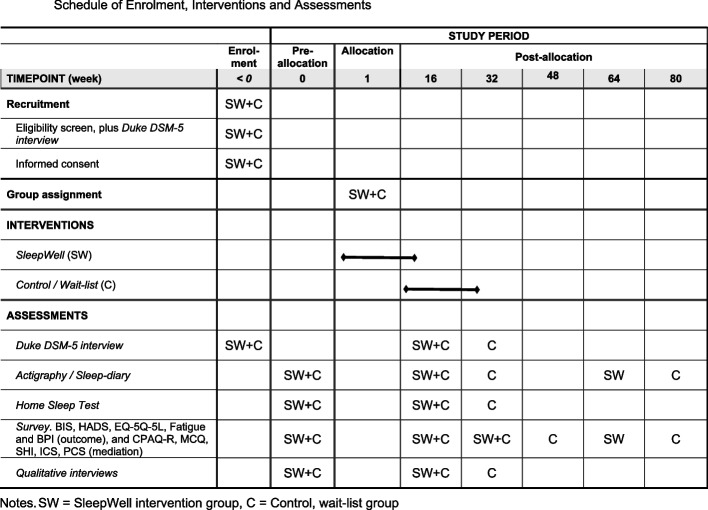
Schedule of enrolment, interventions and assessments. *SW* SleepWell intervention group, *C* control, wait-list group

The timeline for participant recruitment, intervention delivery and assessments is outlined in Figs. 1 and 2. After initial screening and confirmation of eligibility, participants will complete baseline assessments before randomisation. The intervention group will begin the SleepWell programme immediately, while the comparison group will continue TAU. Assessments will occur at the following intervals:

- Baseline (Week 0): Comprehensive evaluations using the Duke sleep interview for insomnia, sleep diaries, actigraphy, PSG/Home Sleep Test REM and self-report psychological instruments related to primary and secondary outcome variables, including mediation variables.
- Post-Intervention (Week 16): Assessment of short-term outcomes in SleepWell group, and second baseline measurement of the TAU group. The measurements are similar to those collected at baseline (week 0), in addition to qualitative interviews with the participants’ experiences of receiving the intervention.
- Post-Intervention for TAU (Week 32): This assessment is conducted after the TAU group has received the SleepWell intervention. The assessments are similar to those used at the post-intervention at week 16.
- Follow-Up Assessments: These are conducted 4 and 12 months after the post-test to evaluate maintenance of treatment effects. This is planned for weeks 32 and 64 in the SleepWell study arm and weeks 48 and 80 in the TAU arm. Self-report data are collected at the first follow-up test. At the final follow-up, both self-report data and actigraphy data are collected.

Participants in the TAU arm will cross over to the SleepWell programme approximately 4 months after the baseline, ensuring access to the intervention while preserving the integrity of the initial randomisation.

### Sample size {14}

A meta-analysis of comparable somatic diagnoses (5 studies) indicated a standardised mean difference (Cohen’s *d*) between intervention and control ranging between 0.60 and 0.80 [18]. With randomisation done on an individual level, a correlation = 0.5 between pre- and post-test (R-sq = 25%), alpha = 0.05, power = 0.80 and minimum Cohen’s *d* = 0.60, at least *N* = 68 patients are required to reject the null hypothesis. With an estimated intra-class correlation (ICC) = 0.05 (adjustment for similarities that occur between patients due to sharing of a treatment group, cluster size = 6), which yields a design effect of 1.25 (1 + (6–1)*ICC), at least *N* = 85 participants are needed. Adding a safety margin of 20% dropout means a final sample estimate of *N* = 106 patients (*n* = 53 SleepWell, and *n* = 53 TAU).

### Recruitment {15}

Patients will be recruited from the Department of Pain Management, and Physical Medicine and Rehabilitation at the University Hospital of North Norway. These two departments evaluate and treat patients with persistent pain who frequently suffer from comorbid insomnia. Medical doctors, clinical psychologists, physiotherapists or nurses will orally inform about the SleepWell study and provide written study/registration information to eligible patients who accept an invitation to participate. Patients may also register via flyers/posters in the clinic waiting rooms. Patients will also be recruited from selected GPs in the city of Tromsø using the study’s inclusion and exclusion criteria. Based on experiences from previous group-based interventions at the same hospital departments, patient dropout is expected. Therefore, we recruit 1–2 extra patients to each study arm to ensure therapy group sizes of > 6 participants who finish.

### Methods: assignment of interventions

#### Sequence generation {16a}

The participants will be block-randomised with a variable block size of 4, 6 or 8 to maintain equal treatment and TAU group sizes and to conceal study arm allocation. The randomisation is performed using the “blockrand” library of RStudio with the following syntax: *randomisation* <—*blockrand*(*n* = *106*, *num.levels* = *2*, *levels* = *c(*“*SleepWell*”, “*TAU*”), *block.sizes* = *c*(*2*,*3*,*4*)) and *print*(*randomisation*).

#### Concealment mechanism {16b}

During the process of obtaining informed consent (item 26a), patients will undergo several assessments, including the collection of survey data via the www.nettskjema.no, a 2-day polysomnography/home sleep testing, and a 7-day sleep diary alongside actigraphy recording. Importantly, both research assistants and patients will remain blinded to the specific study arm to which each patient will be assigned. The principal investigator (PI) will generate the entire randomisation sequence using the above RStudio syntax and inform patients/assistants about the group assignment when at least 12, or preferably 16, patients have been included in the study. Knowledge of the group assignment remains concealed until participants have passed baseline assessments.

### Implementation {16c}

After completing the baseline registrations, a research team member will inform each patient about study arm allocation, and notably, that those allocated to the TAU arm will receive the SleepWell condition after the four-month waiting period.

### Blinding

#### Who will be blinded after assignment to the intervention {17a}

The intervention’s specific characteristics preclude the blinding of therapists and patients. Participating patients will be fully informed about the study to ensure they understand and accept their assigned treatment arm. To prevent drop-out, TAU patients must understand that they will receive the SleepWell treatment approximately four months after their study inclusion (Fig. 1). Data analysts will be blinded by masking the group allocation, as will the research assistants collecting data, until all patients have completed the post-test assessment.

#### Procedure for unblinding if needed {17b}

Since blinding is not implemented in this trial, unblinding procedures are not applicable.

## Methods: data collection, management, and analysis

### Plans for assessment and collection of outcomes {18a}

The *primary outcomes* are measured by the following insomnia indicators:

- The Duke semi-structured clinical interview for deciding the presence of DSM-5 insomnia criteria [60, 61], and the Bergen Insomnia Scale (Five self-report items) for quantifying the dimensional level of DSM-5 insomnia nocturnal symptoms (sleep onset, maintenance and early awakening) and daytime consequences (poorer function in work, school or social life) that have lasted ≥ 3 months and occurred ≥ 3 nights a week [67]. The Duke interview also probes for other sleep disorders regarding patient exclusion, notably restless legs or sleep apnoea.
- Actigraphy (Actiwatch Spectrum Plus, Phillips Respironics) worn on the non-dominant arm, providing estimates of physical activity level, light exposure, SOL, duration of wake time during the nights, WASO and EMA. In addition, sleep continuity is calculated as the proportion of time in bed spent sleeping (SE %: sleep efficiency). Recordings will be performed for 7 days, and an average of the sleep indices will be calculated.
- The sleep diary will be manually completed every morning concerning the last night’s sleep and every evening concerning daytime fatigue or sleepiness. The diary will be used to complement the scoring of the actigraphy recordings.

The *secondary outcomes* are measured by the following:

- A simplified PSG, i.e. the Home Sleep Test REM + (HST) (Somnomedics Randersacker Germany, GmhB), is used to complement actigraphy with sleep architecture assessments at nights 1–3 and REM sleep, arousals and sleep stage shifts. The HST can be used at home, allowing patients to hook up the equipment themselves. The device records 11 signals (3 frontopolar, 2 EOG, 1 EMG, snoring (sound), light in the room, movement, head position, continuous impedance measurement (signal quality), finger plethysmograph with oxygen saturation, heart rate and movement). We will evaluate whether patients receiving the SleepWell intervention attain a higher proportion or longer duration of slow-wave (N3) than light-wave (N1, N2) sleep than TAU patients and whether they attain a reduction in the arousal index or sleep stage index indicative of sleep disturbance. The recording will be made two nights per patient due to the “first night effect” related to habituation [68]. However, since the HST is less intrusive to wear and sleep with at home than a full PSG, the average of both nights will be used.
- The Hospital Anxiety and Depression Scale (HADS, 14 items) is designed for hospital populations as it rates the degree of non-vegetative symptoms of depression (7 items) and anxiety (7 items). Item scores range from 0 to 3, and total scores range from 0 to 21 for each subscale [69]. It is widely used as a screening instrument, and a HADS score ≥ 8 is used as a diagnostic marker [70] in Norway.
- The EQ-5Q-5L is a broad quality-of-life index covering the domains of mobility, self-care ability, daily activity function, pain/discomfort and affective symptoms [71].
- The Chalder Fatigue Scale (11 items) measures the degree of physical (7 items) and mental exhaustion (4 items) [72]. Each item is scored from 0 to 3 (total range, 0–33), with higher scores indicating more fatigue.
- The Brief Pain Inventory (BPI, 11 items) indexes pain intensity and interference, as well as body localisation of pain [73].

The measurement instruments for the mediation analysis are:

- Pain acceptance is measured using the Chronic Pain Acceptance Questionnaire (CPAQ-R, 20 items) [74]. Dysfunctional Beliefs and Attitudes of Sleep (16 items) surveys negative cognitions about sleep (i.e. worries about getting too little sleep, helplessness, negative sleep expectations and worries about sleep medication) [75].
- The Meta-Cognitions Questionnaire–Insomnia (15 items) indexes metacognitions related to insomnia, i.e. attention towards and intrusive thoughts about lack of sleep [76].
- The Sleep Hygiene Index (13 items) measures the loss of sleep pressure during the day, e.g. lack of sleep routines, mapping, evening physical activity, use of stimulants or poor sleeping conditions [65].
- The Insomnia Catastrophizing Scale (20 items) indexes arousing thoughts about the consequences of lack of sleep [21].
- Pain Catastrophizing Scale (13 items) [77] assesses worst-case thinking about pain related to rumination, exaggeration and helplessness [78].
- The Hyperarousal Scale (26 items) measures the degree of hyper-activation and discriminates several types of psychophysiological insomnia activation [79].

One research member (KT) conducts the Duke semi-structured clinical interview via telephone. All questionnaire data are collected using *Nettskjema*, which is an online and secure, GDPR-approved tool for collecting questionnaire data through a service developed by the University of Oslo (https://nettskjema.no/). Participants receive a link to the survey and complete it electronically using a smartphone, tablet or laptop. Participants receive the sleep diary, the actigraphy and the simplified PSG devices from the research assistant during visits to patients’ homes. The data collection occurs on the same day that participants receive the first link to the survey, ensuring that all measurements are taken simultaneously. The sleep diary is a paper-based tool, and is manually entered into the study database.

The qualitative, semi-structured interviews will be conducted to explore the feasibility and acceptability of the intervention from the participants’ perspective. To ensure variability in the participants’ experiences, we plan to use self-reported insomnia symptoms at post-test to invite a comparable number of patients with varying treatment responses, ranging from favourable to less favourable. Patients who drop out or withdraw during the intervention will also be invited to participate in the interviews. The interviews will be in person or via telephone, based on the participant’s preferences. The Nettskjema service also includes a dictation application that will be used to audio record the interviews before they are transcribed verbatim for analysis.

### Plans to promote participant retention and complete follow-up {18b}

During recruitment, patients will receive detailed information about the purpose of the study, the randomisation and the requirements related to the intervention. To promote patient retention, baseline registrations will be conducted before the randomisation. Using the “*Nettskjema*” survey platform to collect the questionnaire data may provide a level of flexibility and convenience for the participants, thus reducing the risk of missing data. Nevertheless, patients considered eligible and who consent to participate have the option of unconditional withdrawal; if so, they will suffer no consequences for further medical treatments. In line with the intention-to-treat approach, patients who drop out or withdraw from the study after collecting baseline data will be included in the final analyses unless they have withdrawn their consent. Patients who drop out or withdraw during the intervention will also be invited to participate in the planned qualitative interviews to explore their experiences of treatment feasibility and acceptability.

### Data management {19}

The study complies with the General Data Protection Regulation (Ch. 2, Art. 6/pt. 1e and Art. 9/pt. 2j) and the institutional data management and storage guidelines set by UiT–The Arctic University of Norway. All patient data will be stored in a de-identified form. Data collected during a night’s sleep by the Home Sleep Test REM + device will be uploaded to a web-based service by Somnomedics (using Microsoft Azure server setup) for automated scoring of the polysomnogram, thereafter downloaded to the Microsoft Office365 Sharepoint service hosted by the UiT–The Arctic University of Norway for revision by a clinical neurophysiologist/somnologist. The actigraphy recordings will be uploaded directly to the UiT Office365 Sharepoint service. Survey- and qualitative data are collected/registered through https://nettskjema.no and will be managed digitally using the Service for Sensitive Data (TSD) (a secure service for sensitive data managed by the University of Oslo), which ensures data integrity. After completing the data collection process, the data are transferred to the UiT Microsoft Office365 Sharepoint server. Data access is restricted to the PI, who may authorise access to project members. The Sharepoint service has a 30-day file version history with regular backup routines.

### Statistical methods for primary and secondary outcomes {20a}

Patients randomly assigned to the waiting list (WL) group will conduct two baseline measurements separated by ~ 4 months, with the second one serving as the comparator to the post-test for the SleepWell groups. WL patients then cross over to the SleepWell intervention (Fig. 1) and follow the same plan for post-test and follow-up measurements as the SleepWell study arm.

The primary hypothesis examines the mean group difference between the SleepWell post-test status for all patients receiving treatment and the second baseline status of the WL patients having only received TAU. Since this test involves only two repeated measurements, a conventional analysis of covariance is used with the baseline value of the outcome added as a covariate. Designating baseline values as covariates has become standard practice in therapy research as it improves the precision of the mean difference estimate [80], which also improves the statistical power [81] in estimating the hypothesised mean group difference (0-SleepWell, 1-TAU).

Generalised linear mixed regression models are preferred due to the flexibility in managing most variables (continuous, binary, counts, etc.) and data clustering. Here, some degree of clustering may occur between individuals in the same therapy group as they share a common therapy climate (fellow group patients and therapists), which may underestimate the standard errors and increase the risk of a type 1 error in hypothesis testing. The effect of this nesting will be managed by adding the group number as a random factor.

The GenLinMixed models for the later follow-up tests are comparable, except they add a random intercept factor in addition to the baseline adjustment to account for any leftover correlations in the repeated data, which adjusts the error bands appropriately. Because all patients have received treatment at follow-up, direct testing of general treatment–control effects at follow-up is irrelevant (the *Group* factor). The estimable fixed factors thus become *Time* (1-post, 2-follow-up at 4 months and 3-follow-up at 12 months) and the Time*Group interaction that allows mean group differences at different time points. We will analyse if relapse occurs during the follow-up period by conducting repeated time series contrast analyses. In case of significant contrasts indicative of relapse, we will examine for possible differences between the SleepWell (early treatment) and the TAU (late treatment) study arms. Effect size statistics will be given as a standardised mean difference by dividing the beta-weight for a group comparison or a time difference with the within-subject standard deviation for the test, i.e. the residual SD.

The analyses will be conducted conservatively as intention-to-treat. Alpha values < 0.05 and 0.01 are assumed for the hypotheses about primary sleep treatment outcomes, i.e. SOL, WASO, SE and EMA, and secondary outcomes, i.e. affective health, fatigue and quality of life, respectively. The alpha value is set to 0.01 for analyses of moderators and mediators. The GenLinMixed handles missing data adequately without the need for multiple imputations, as it estimates the parameters based on all data available [82], thus reducing biases related to dropout given a missing-at-random (MAR) dropout pattern.

Since moderator variables in CBT-i are imperfectly understood [83], exploratory moderator analysis will extend the above regression models by adding the moderator variable with its two-way interactions with Time and Group to get a full three-way interaction that may discern exactly the subgroups defined by the moderator that benefit the most from treatment.

### Methods for additional analyses {20b}

Additional analyses serve the purpose of exploring the most effective intervention elements. This will be carried out by using the variables collected for mediation analysis to estimate possible mediation effects. Moreover, to explore the feasibility, relevance and acceptability of the intervention and its separate parts (Table 1), additional analyses include qualitative, semi-structured interviews. We seek to identify the major conceptual dimensions that meaningfully account for all the interview texts, by analysing transcribed interview data using the reflexive thematic analysis approach [84, 85]. The analytic process is dynamic, moving between six analytic phases: (1) reading of the interviews to obtain an overview; (2) marking of text relevant to the research question; (3) sorting text into broader thematic groups; (4) reviewing of thematic text groups for coherence, consistency and meaning related to the research question; (5) definition and naming of themes that capture the meaning of the data; and (6) a final synthesisation. The COREQ criteria for reporting qualitative research will be applied [86]. Patients are eligible for such interviews, whose primary sleep outcome measure shows negligible, and favourable treatment effects, respectively. In each of these subgroups, the number of patients needed to reach data saturation will align with guidelines from a recent systematic review [87].

### Methods in analysis to handle protocol non-adherence and any statistical methods to handle missing data {20c}

The GenLinMixed analysis is favourable for data missing at random as it estimates the parameters based on all available patient data. This reduces biases related to the expected dropout, given that a pattern of missing at random can be presumed. If covariates in the study are correlated with missingness, adjustment for missing is possible by including these as regression covariates.

## Methods: monitoring

### Composition of the data monitoring committee, its role and reporting structure {21a}

A data monitoring committee is not considered relevant for the present study.

### Interim analyses {21b}

No interim analyses are planned for this study. The SleepWell intervention is not expected to cause harm, eliminating the need to arrange stopping procedures to terminate the trial.

### Adverse event reporting and harms {22}

There is no evidence that the intervention may cause harm or serious adverse effects. However, the weekly group sessions with the patients enable continuous monitoring of the study participants and to uncover any negative events from participating in the SleepWell intervention.

### Frequency and plans for auditing trial conduct {23}

There are no planned study audits.

### Plans for communicating important protocol amendments to relevant parties, e.g. trial participants, and ethical committees {25}

Any protocol amendments will be forwarded to the ethics committee for approval before implementation.

### Who will take informed consent? {26a}

Written informed consent is mandatory for study participation, and patients may unconditionally withdraw their consent at any time. Participants are given oral and written information about the trial and a QR code/link to access additional information and the consent form, and they provide their consent by electronically signing the form.

### Additional consent provisions for collection and use of participant data and biological specimens {26b}

Additional consent is not relevant for this study.

### Confidentiality {27}

To ensure secure data storage, all patient data will receive an Azure Information Protection (AIP) classification as “confidential” in the UiT Office365 Sharepoint service. Data are encrypted with AES 256-bit encryption keys. All patient data are stored with a unique ID number to avoid personal identifiers. The linkage key connecting the ID numbers with patients’ contact information is encrypted and stored elsewhere. The linkage key is only accessible to the PI. The linkage key will be permanently deleted after the defined project end date (Dec 2030) as devised by the Regional Committee for Medical and Health Research Ethics, which approved the study. Future publications or public disseminations will not disclose personal information about the study participants, and published results will be presented in a manner that prevents re-identification of patients.

### Ancillary and post-trial care {30}

Norwegian patients are covered by the “Norwegian Patient Injury Compensation” arrangement. Since the risk of serious adverse events following participation in the SleepWell intervention is improbable, we do not plan for post-trial care. Measures to compensate for any harms associated with trial participation thus appear irrelevant; hence, there are no plans to establish stopping procedures to terminate the trial or to provide ancillary or post-treatment care. Patients who drop out or do not respond to the SleepWell intervention will be encouraged to contact their GP for further assessment and possible sleep treatment.

### Dissemination plans {31a}

At least 5–6 academic papers can be expected, which will be submitted to high-impact and open-access journals, preferably to pain and sleep disorder-related journals. Findings will also be disseminated at national and international scientific conferences on sleep disorders, pain management and behavioural interventions. Lay summaries will be shared with patient support groups and community health organisations to ensure accessibility for non-academic audiences.

### Authorship eligibility guidelines and any intended use of professional writers {31b}

No publications generated from the study will in whole or in part be produced using artificial intelligence assistance or professional writers. The Vancouver Declaration will guide decisions on authorship eligibility.

### Plans to give public access to the full protocol, participant-level data and statistical code {31c}

There will be no restrictions on public access to the complete protocol.

### Plans for collection, laboratory evaluation and storage of biological specimens for genetic or molecular analysis in this trial/future use {33}

This is not applicable because no biological specimens will be collected.

## Discussion

This protocol outlines an RCT study hypothesising that a cognitive therapy approach (the SleepWell intervention) to treat patients with comorbid sleep problems and persistent pain will perform better than TAU concerning the primary outcome of reducing DSM-5 insomnia symptoms. We also expect the intervention to perform better than TAU using a battery of secondary outcome measures, which comprise subjectively reported pain-related symptoms, the need for sleep medication, fatigue, anxiety, depression and quality of life, as well as objectively measured sleep indicators.

Considering the limited number of controlled trials, this study may contribute to filling several knowledge gaps in the sleep therapy literature on comorbid insomnia and persistent pain. First, the present study adds to the literature by focusing on both subjective and objective primary and secondary outcomes. The use of sleep questionnaires, actigraphy- and polysomnography-based sleep measures provides good additional opportunities for examining the role of discrepancies between subjective and objective sleep measures [24]. Secondly, we will provide detailed information about the nature of the intervention, notably its meta-cognitive elements, and the measuring of treatment manual fidelity. Third, the present study aims to be detailed in the search for effects. From the available literature reviews [18, 38, 88], it should be of no surprise to find that the SleepWell intervention will perform better than the TAU control condition because the latter represent an unsystematic and an insufficient degree of health service. The detailed search for effects will focus on the moderator analyses. Such analyses are suitable for exploring sample differences in how the Sleep-Well intervention performs, i.e. whether specific subgroups of eligible patients benefit more from the treatment than others. Additionally, qualitative interviews will be performed among participants who respond favourable to the intervention, and among those who do not or even drop out during the intervention. Such interviews may provide a better understanding of participants’ experiences taking part in sleep intervention studies about their feasibility, relevance and acceptability. This information can help identify potential inconsistencies that might need adjustments to the intervention, particularly in cases where the intervention is successful. Still, participants meet practical challenges that could be addressed through manual or procedural modifications. Such interviews may also contribute to detecting whether patients’ experiences of particular treatment elements elicit changes in how the intervention is conducted. Finally, and for the same purpose, we will also use mediation analyses to identify the most salient possible treatment mechanism underpinning a significant treatment effect. Here, we expect that the meta-cognitive elements may perform equally or better than the cognitive elements; furthermore, these effects will be compared to the behavioural variables included in the study.

### Study limitations

There are some limitations to be considered. First, no other evidence-based treatments are available for patients suffering from the insomnia-pain comorbidity aside from CBT-i. Considering the marginal effects of TAU [26–28], TAU as a comparison condition may not be an optimal test for the effectiveness of the SleepWell intervention. Second, a recent meta-analysis has found that sleep disturbances may impair pain inhibition among females but not among men [89], yet possible sex differences in effects are not examined in the present study. Third, the study’s reliance on a group therapy format may introduce a selection bias. Patients who are uncomfortable with sharing personal issues in a group setting, especially those with more severe medical or psychological conditions, may be less likely to participate, potentially limiting the generalisability of the findings. Furthermore, face-to-face participation may exclude participants with long travel distances to the study site. If so, some selection bias may be introduced in terms of overestimating treatment feasibility, but not in terms of clinical severity and treatment outcome.

## Conclusion

The SleepWell intervention represents a promising strategy for addressing the dual burden of insomnia and persistent pain. If the primary hypothesis about its effectiveness is supported, SleepWell could significantly advance clinical management by providing a structured, evidence-based alternative to the current treatment approaches, which often rely on sleep medications and unsystematic psychoeducation.

Beyond its direct benefits, this study aims to provide critical insights into the mechanisms underlying CBT-i’s effectiveness. By exploring both subjective and objective outcomes and incorporating patient perspectives, the findings will inform the development of more tailored and effective interventions. Findings may thus shape clinical guidelines and improve the quality of care for this patient population.

### Trial status

The study recruits participants from January 2025 and estimates to complete the inclusion within December 2026.

## Data Availability

Upon scientifically reasonable requests, anonymised patient data may be made available to third parties 5 years (Dec 2035) after the project has reached the registered finishing date (Dec 2030) as approved and required by The Regional Health Authorities of Northern Norway. Examples of such requests may relate to combinations of data sets, providing data for meta-analyses, a re-analysis to secure good scientific practice, or requests to conduct new data analyses.

## Abbreviations

BIS: Bergen Insomnia Scale
CAP: Cyclic alternating patterns
CBT-i: Cognitive Behavioural Therapy for insomnia
COREQ: Consolidated Criteria for Reporting Qualitative Research
DPIA: Data Protection Impact Assessment
EMA: Early Morning Awakening
GP: General practitioner
HST: Home Sleep Test
IASP: International Association for the Study of Pain
ICC: Intra class correlation
MAR: Missing at random
MCT: Meta cognitive therapy
PI: Principal investigator
PSG: Polysomnography
REM: Rapid eye movement
SE: Sleep efficiency
SOL: Sleep onset latency
TAU: Treatment as usual
TSD: Service for Sensitive Data
TST: Total sleep time
WASO: Wake After Sleep Onset
WL: Waiting list
